# Inhalational Anesthesia for Near-fatal Pediatric Asthma Complicated by Malignant Hyperthermia

**DOI:** 10.7759/cureus.19032

**Published:** 2021-10-25

**Authors:** Davij Pasrija, Justin Assioun, Mohammad Sallam, Andrew Prout

**Affiliations:** 1 Department of Pediatric Critical Care, University at Buffalo, Buffalo, USA; 2 Department of Pediatrics, University at Buffalo, Buffalo, USA

**Keywords:** asthma, isoflurane, malignant hyperthermia, extracorporeal gas exchange, inhaled anesthetic agents

## Abstract

Acute severe asthma is a commonly encountered condition in the pediatric emergency room and the pediatric intensive care unit (PICU). Its treatment involves the use of bronchodilatory agents acting on different receptors, steroids to reduce ongoing inflammation, and non-invasive or invasive mechanical ventilation to offload the increased work of breathing from the respiratory muscles. Patients refractory to these therapies may require the use of inhaled anesthetic agents and extracorporeal gas exchange (ECMO) for life-threatening asthma exacerbations. Depending on institutional protocols, the use of these therapies may vary. The use of inhaled anesthetic agents for asthma management in the PICU is infrequent and is limited to centers with specialized equipment. Commonly encountered side effects include hypotension, arrhythmias, and delirium. Malignant hyperthermia (MH) is a well-known but infrequent side effect of inhaled anesthetic use, depolarizing muscle agents, and has not been described in the PICU following the use of anesthetics for pediatric asthma.

## Introduction

Patients with severe asthma exacerbations form a significant subset of patients admitted to the pediatric intensive care unit (PICU). Initial treatments include bronchodilator agents like β2 agonists (salbutamol and terbutaline) administered via inhalation, intravenously, or subcutaneously. Anticholinergic agents like ipratropium and tiotropium (which is often used in adults with chronic obstructive pulmonary disease, COPD) reduce mucous secretion from cells lining the airways and cause bronchial smooth muscle relaxation. Steroids form the mainstay of treatment to stop and prevent further airway inflammation. Other second-line agents include the use of magnesium sulfate, ketamine, and methylxanthines like aminophylline or theophylline, all of which cause bronchial smooth muscle relaxation by different mechanisms. A significant number of patients with severe exacerbations may require non-invasive positive pressure ventilation (bi-level non-invasive positive airway pressure, BIPAP/continuous positive airway pressure, CPAP) to help with increased work of breathing [1]. If the patient does not improve with the above-mentioned therapies, they will often require intubation and mechanical ventilation. In cases of refractory and life-threatening bronchospasm, despite adequate sedation and muscle relaxation provided during mechanical ventilation, inhaled anesthetic agents and extracorporeal gas exchange (ECMO) [2] have been used as rescue therapies. The use of anesthetic agents is based on institutional availability and protocols. They are delivered via specialized ventilatory circuits which are commonly used in the operating room (OR). Treatment with inhaled anesthetics outside of the OR settings is uncommon given the potential for serious complications, as well as the need for specialized equipment and trained personnel [3]. Isoflurane therapy for near-fatal asthma has been successfully utilized in few PICUs across the United States. The most common side effect currently encountered is systemic hypotension and the emergence of delirium being the other major challenge. The incidence of malignant hyperthermia (MH) ranges from 1:10,000 to 1:250,000 anesthetic procedures as per different studies [4]. It is a well-documented but rare complication following the use of inhaled anesthetic agents, depolarizing muscle blockade use, and other triggers. Its pathophysiology involves the uncontrolled release of calcium from the sarcoplasmic reticulum, most commonly secondary to a defective calcium channel known as the ryanodine receptor, although other mutations can be involved [4]. The onset of MH is usually within a few minutes to hours after exposure to an inciting agent. Its diagnosis is based on both clinical and laboratory features which include excessive tachycardia, rise in end-tidal carbon dioxide levels despite the increase in minute ventilation, elevated body temperature (more than 38.1 °C), muscle breakdown as evidenced by elevated creatine kinase levels usually more than 10,000 units/ml. There is a scoring system based on these features to determine the likelihood of malignant hyperthermia, ranging from almost never to almost certain [5].

## Case presentation

An 11-year-old female with a history of moderate persistent asthma presented with a severe asthma exacerbation requiring PICU admission. Respiratory viral panel with PCR testing for common viral triggers including rhinovirus, influenza A/B, metapneumovirus, and eight other viruses was negative. Viral testing did not include COVID 19 testing and was prior to COVID 19 pandemic. The patient was started on conventional first-line asthma therapy, including inhaled and intravenous (IV) β2 agonists, IV methylprednisone, and a theophylline infusion. A couple of hours after transfer, the patient continued to have poor aeration and use of accessory muscles of breathing. Intermittent doses of IV ketamine and magnesium sulfate were tried with no significant improvement. The patient was started on non-invasive ventilation (BIPAP) which was escalated to pressures of 20/10 (IPAP/EPAP). Despite the escalation in therapeutic modalities, the patient continued to be in significant respiratory distress and providing blood gas notable for hypercapnia (pH - 7.24/58 (carbon dioxide)/−4.5 base deficit). A decision was made for endotracheal intubation after six hours of presentation to the hospital. Mechanical ventilation was continued for the next two hours with tidal volumes of 9-10 ml/kg and respiratory rates reduced to as low as seven to eight breaths per minute to allow for adequate time for exhalation and carbon dioxide clearance. Sedation was optimized with fentanyl, dexmedetomidine, and a cisatracurium infusion was used for continued smooth muscle relaxation. Despite these interventions, the patient continued to have increased carbon dioxide levels on arterial blood gas (7.04/85/-8.1). At this point in time, a decision was made to start the patient on inhaled isoflurane in view of worsening respiratory and metabolic acidosis. A few hours after starting isoflurane, blood gases improved (ph- 7.28/57/−3.5). Six hours into inhaled anesthetic therapy the patient developed persistent fevers with a max temperature of 41 °C, worsening tachycardia (HR 180s from 130s previously), and metabolic acidosis accompanied by a rise in arterial PCO_2_ (ph - 6.97/105/−15.3 mmHg from previous carbon dioxide levels of 64 mmHg). Serum creatinine kinase (CK) levels which had been normal at admission were measured to be >12,000 units/ml, urinary myoglobin was positive. Creatine kinase levels peaked at >30,000 units/ml over the next 24 hours and improved later with increased hydration and urinary alkalization. As per the earlier mentioned scoring system, the patient had a clinical MH score of 48, placing her in the very likely category.

Isoflurane was promptly discontinued after discussion with the anesthesiology team and IV dantrolene was started for treatment of MH. The patient was planned for urgent veno-venous (VV) ECMO cannulation due to the return of severe bronchospasm after isoflurane discontinuation. Dantrolene was continued every eight hours for the next three days although body temperature and metabolic acidosis began to improve within one to two hours after discontinuation of isoflurane. In this case, genetic testing was not performed due to rapid improvement in symptoms after discontinuation of isoflurane and the high likelihood of MH based on Larach score. The patient did require ultrafiltration through the ECMO circuit for excessive fluid removal, however, she had no evidence of renal dysfunction based on blood urea, creatinine, and electrolyte disturbances. Significant improvement in bronchospasm was noticed based on blood gas on mechanical ventilation, despite discontinuation of gas flow through ECMO machine. The patient was then successfully decannulated on day 5 of the ECMO run and was successfully extubated two days later. The remainder of the hospital stay was uneventful and the patient was ultimately discharged home with an intact neurological status.

## Discussion

Few case series and case reports have discussed the use of inhaled anesthetics, such as sevoflurane and isoflurane, with regards to the treatment of refractory asthma [6]. One study out of a single, tertiary care PICU evaluating isoflurane use in pediatric asthma described systemic hypotension, arrhythmia, and pneumothoraces as its most common complications [7]. Isoflurane and sevoflurane are more commonly used to treat pediatric asthma as desflurane has been shown to adversely affect respiratory system dynamics [8]. None of these studies have previously identified MH as a complicating factor during inhaled anesthetic use for pediatric asthma. As previously mentioned, MH is a rare complication following inhaled anesthetic use and is more commonly seen with depolarizing muscle relaxation agents. The variability in the onset of clinical symptoms and lab values makes the diagnosis of MH difficult. Traditionally, the onset of MH is known to be 30 minutes to 1.5 hours after exposure to the inciting agent with the most rapid onset shown to be after halothane (20±5 minutes) compared to isoflurane (48±24 minutes) [9]. Interestingly in our case, signs and symptoms of MH began to manifest approximately six hours after isoflurane initiation.

## Conclusions

As per our review of the literature, this is the first reported case of MH following the use of isoflurane for pediatric asthma in the PICU. Given the infrequent use of inhaled anesthesia in the PICU and the secondary occurrence of MH, it is critical for PICU providers to have a high index of suspicion for this adverse effect. It is also important to keep in mind that the onset of this complication can occur many hours after initiation of therapy. Patients with asthma can have rebound bronchospasm after sudden discontinuation of inhaled anesthetic therapy and clinicians should be prepared for other rescue therapies like extracorporeal support for maintaining gas exchange.
